# PTH1R Actions on Bone Using the cAMP/Protein Kinase A Pathway

**DOI:** 10.3389/fendo.2021.833221

**Published:** 2022-01-19

**Authors:** T. John Martin

**Affiliations:** Department of Medicine, St Vincent’s Institute of Medical Research, St Vincent’s Health, University of Melbourne, Fitzroy, VIC, Australia

**Keywords:** PTH, PTHrP, osteoblasts, osteoclasts, adenylyl cyclase, cAMP, protein kinase A

## Abstract

After the initial signaling action of parathyroid hormone (PTH) on bone was shown to be activation of adenylyl cyclase, its target was found to be cells of the osteoblast lineage, to the exclusion of osteoclasts and their precursors. This led to the view that the osteoblast lineage regulated osteoclast formation, a proposal that was established when the molecular mechanisms of osteoclast formation were discovered. This is in addition to the effect of PTH1Rv signaling throughout the osteoblast differentiation process to favour the formation of bone-forming osteoblasts. Initial signaling in the PTH target cells through cAMP and protein kinase A (PKA) activation is extremely rapid, and marked by an amplification process in which the later event, PKA activation, precedes cAMP accumulation in time and is achieved at lower concentrations. All of this is consistent with the existence of “spare receptors”, as is the case with several other peptide hormones. PTH-related protein (PTHrP), that was discovered as a cancer product, shares structural similarity with PTH in the amino-terminal domain that allows the hormone, PTH, and the autocrine/paracrine agent, PTHrP, to share actions upon a common G protein coupled receptor, PTH1R, through which they activate adenylyl cyclase with equivalent potencies. Studies of ligand-receptor kinetics have revealed that the PTH/PTH1R ligand-receptor complex, after initial binding and adenylyl cyclase activation at the plasma membrane, is translocated to the endosome, where adenylyl cyclase activation persists for a further short period. This behavior of the PTH1R resembles that of a number of hormones and other agonists that undergo such endosomal translocation. It remains to be determined whether and to what extent the cellular effects through the PTH1R might be influenced when endosomal is added to plasma membrane activation.

## Introduction

The adenylyl cyclase (AC) complex is an essential component of information transfer to the interior of cells. Under the influence of specific receptor-related events it catalyses the formation of cAMP, a process that is regulated by either stimulatory or inhibitory guanine nucleotides (1). A wide range of intracellular processes are influenced by cAMP, mainly through activation of cAMP-dependent protein kinase (PKA) (2). In this manuscript the initial actions of parathyroid hormone (PTH) on cAMP through the PTH1 receptor (PTH1R), a G protein-coupled receptor (GPCR), will be considered, with the aim of relating these to later events in target cells. Early work describing the effects and attempting to determine molecular mechanisms will be discussed, as well as the conclusions from those studies and their how they fit into modern concepts developed as a result of new methods of studying the trafficking and actions of proteins in cells.

## PTH Actions on the Cells of Bone

Soon after discovery of the importance of cAMP in signal transduction, the stimulation of adenylyl cyclase (AC) and generation of cyclic AMP (cAMP) in response to parathyroid hormone (PTH) were demonstrated *in vitro* in rat bone (3) and in kidney (4). Direct effect of a parathyroid - derived substance on bone had been shown when pieces of parietal bone transplanted to the cranial cavity of mice, together with parathyroid tissue, underwent resorption on the side towards the parathyroid (5). When ultimately PTH was purified and active peptides synthesized, ample evidence was obtained from organ culture that PTH stimulates bone resorption (6, 7). Together with the bone resorptive effect of dibutyryl cAMP *in vitro* (8), these and other findings pointed to a role of cAMP in mediating the resorptive action of PTH on bone. For example, both PTH and prostaglandins increased cAMP production in osteoblastic cells, and exogenous cAMP analogues, phosphodiesterase inhibitors and cholera toxin reproduced the resorbing actions of each of these (9, 10).

It was noted that the initial actions of PTH were indeed on the osteoblast lineage, from which bone is formed, without any evidence of direct action on osteoclasts, that are derived from hemopoietic precursors and are responsible for the resorption of bone. Nevertheless the stimulation of osteoclastic resorption by PTH *in vitro* and *in vivo* was beyond doubt. Studies in isolated bone cell populations and in osteosarcoma cells that are phenotypically osteoblastic, showed repeatedly that as well as PTH, the best characterized bone resorbing factors (e.g. prostaglandins. IL-6, IL-1. 1,25-dihydroxyvitamin D_3_), had receptors and/or direct responses in osteoblastic cells. This lack of direct action on osteoclasts by analogs of PTH and several prostaglandins and their metabolites (11–13) continued to be noted when it became possible to study freshly isolated osteoclasts *in vitro* (14). These findings provided the basis for the hypothesis that cells of the osteoblast lineage might be the first point of action of the bone resorbing hormones (12, 15, 16), and that the stimulation of osteoblast lineage cells results in activities that increase the number and activity of osteoclasts (17)

Evidence in support of this hypothesis was gathered over the next several years. First to be established was that bone resorbing agents stimulate resorption by a mechanism that required the presence of osteoblastic cells that made contact with the osteoclast precursors (14, 18, 19). Most importantly, the formation of osteoclasts under the influence of bone resorbing agents was also shown to require contact between osteoblast lineage cells and hemopoietic precursors of osteoclasts. This was achieved using a simple experimental design, of growing osteoblast-rich cells from mice together with osteoclast precursors from bone marrow or spleen either together on the same surface or separated by a cell-impermeable filter (20). The introduction of the filter blocked osteoclast formation (20).

The understanding of cell communication processes revealed by this work over several years, together with the methods that were developed, led to the discovery of osteoprotegerin (OPG), a soluble member of the TNF receptor superfamily expressed by the *Tnfrsf11b* gene, revealing it as a powerful inhibitor of osteoclast formation expressed by osteoblasts (21, 22). This provided the means of identifying and cloning of the TNF ligand family member, Receptor Activator of Nuclear Factor κB Ligand (RANKL) (*Tnfsf11)* (23, 24) that is the essential mediator of osteoclast formation and activity, and production of which is increased by activation of the PTH1R (25, 26). RANKL binds to its receptor RANK (*Tnfsrf11a*), on osteoclast precursors, thereby initiating signaling essential for osteoclast differentiation. The decoy receptor, OPG, has an essential physiological role as a paracrine inhibitor of osteoclast formation, produced by the osteoblasts and binding RANKL to limit its activation of osteoclast formation. Thus it was established and confirmed that the cAMP/PKA signaling pathway mediated the resorptive action of PTH through stimulation of RANKL production *via* the PTH1R in appropriate cells in the osteoblast lineage (27).

Subsequently it emerged that cAMP/PKA signaling was also the major regulatory pathway mediating the anabolic action of PTH. It had been shown many years earlier by Selye (27) and Albright (28) that in contrast to its resorptive effect, when PTH was given by daily injection to animals it had an anabolic effect on the skeleton. These observations were eventually revived in a study in human subjects (29) that foreshadowed the double-blind clinical trial that led to approval for osteoporosis therapy in several countries of PTH (1–34) (teriparatide) for the treatment of osteoporosis (30). Approvals were also obtained somewhat later for clinical use of PTH (1–84) and for an analog of PTH (abaloparatide) (31).

**Figure 1:**
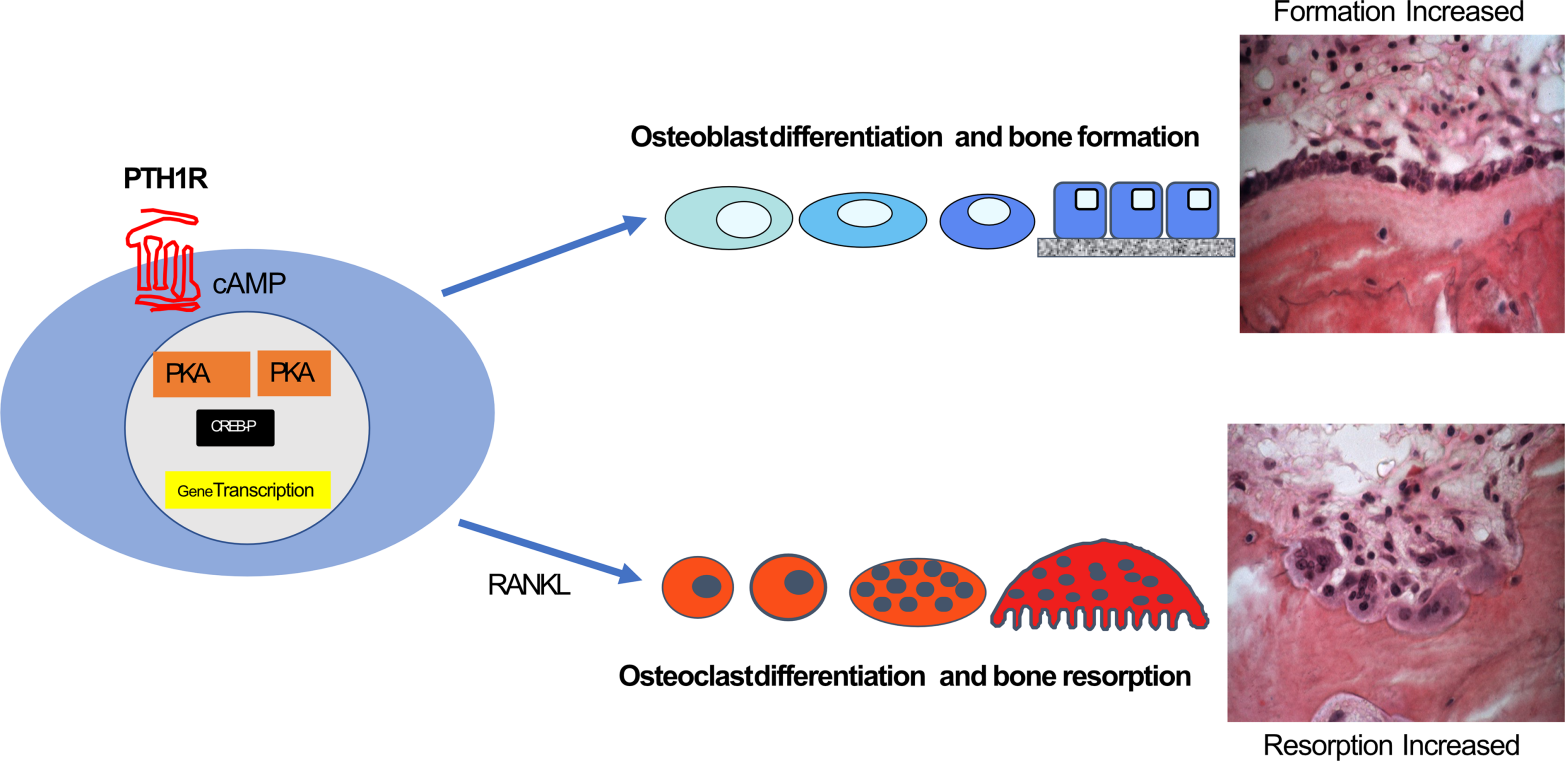
cAMP/PKA/CREB regulation in the osteoblast lineage through PTH1R results in directions along two main pathways. These can be direct effects on osteoblast differentiation and activity throughout the osteoblast lineage leading to increased bone formation, and indirect effects on osteoclast formation from precursors and their activity, mediated by RANKL production (see text).

Several lines of evidence suggested that cAMP/PKA signaling was also central to this anabolic effect (e.g (32, 33). Analogs of PTH with restricted signaling capacities were used to indicate that the dominant pathway for the PTH anabolic effect through its receptor is cAMP/PKA signaling (34). The anabolic skeletal effect through PTH1R signaling requires that the agonist be delivered intermittently (usually daily), reaching a peak in the circulation within an hour and declining to baseline within 3 hours (35–37). Thus, while short daily exposure to PTH1R stimulation favours an anabolic effect on bone cells that entails osteoblast differentiation and bone formation, when PTH1R stimulation is maintained by infusion, high dose, or by rapidly repeated injections, this results in osteoclast formation and bone resorption. Thus the production of cAMP through activation of the PTH1R in cells of the osteoblast lineage results in specific outcomes in two different cell lineages. One is a direct effect to favour osteoblast differentiation, beginning early and acting throughout the mesenchymal osteoblast lineage, the other is an indirect effect on the hemopoietic lineage mediated predominantly by increased production of RANKL and decreased production of OPG in osteoblast lineage cells that are near to hemopoietic precursors of osteoclasts (**Figure 1**).

## Early Events in cAMP and Protein Kinase A Activation in Osteoblasts

The realization that control of cAMP early in the cell response in the osteoblast lineage is a key event in both the resorptive and the anabolic response of PTH required detailed analysis of these early events in cell activation.

**Figure 2:**
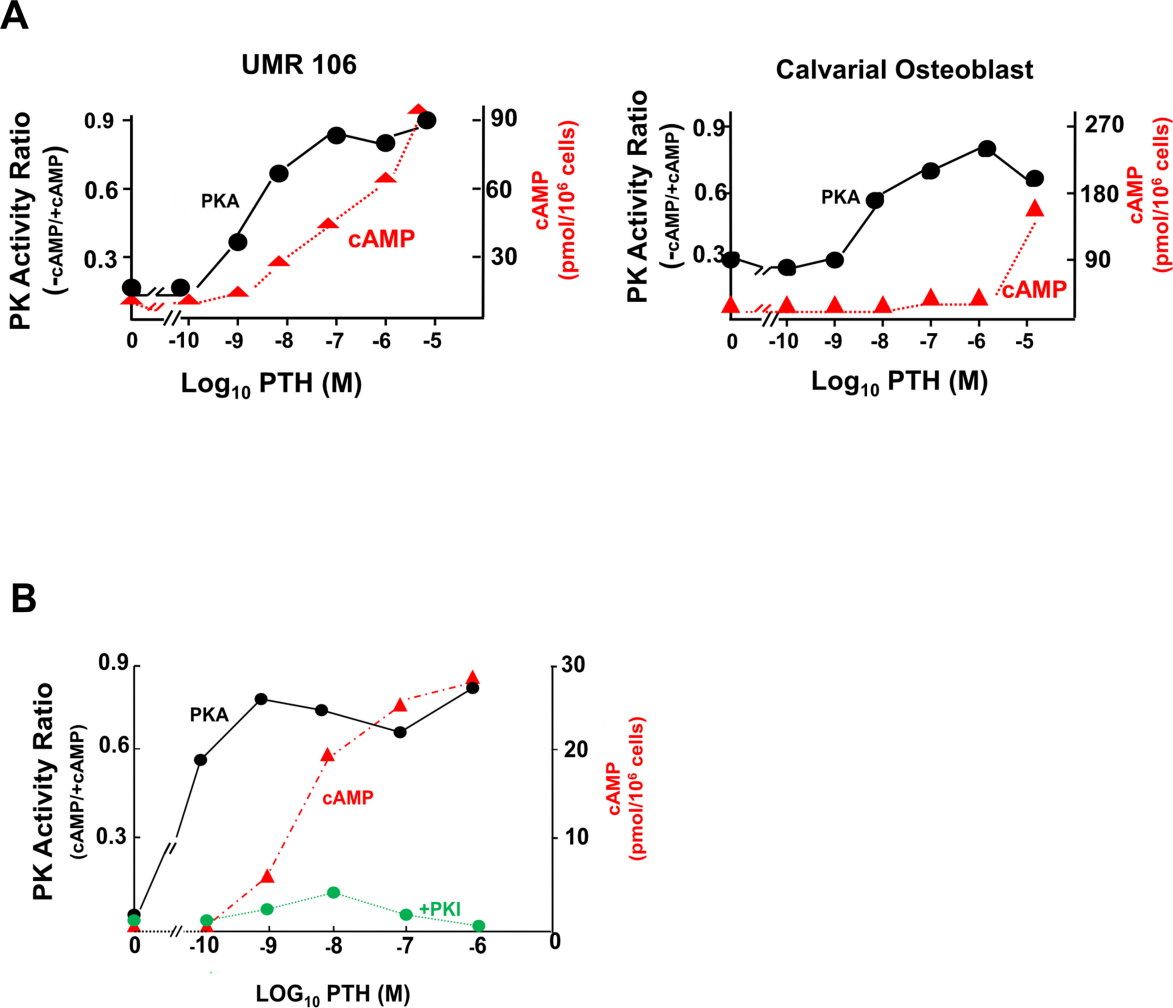
Total cellular cAMP (in red) and activation of PKA (in black) in UMR106 osteogenic sarcoma cells and primary mouse calvarial cells. **(A)** dose-responsive effects of PTH after 60 seconds. **(B)** Activation of PKA by PTH (1–34) in relation to cAMP generation at 60 seconds, and specific inhibition of phosphorylation by heat-stable protein kinase inhibitor (PKI) (from (42) with permission).

It was considered that the major, if not the only, mechanism of cAMP action in mammalian cells was through stimulation of cAMP – dependent protein kinase A (PKA). We therefore focussed on this connection by studying PTH action on osteoblast-rich cultures from newborn rat calvaria and on clonal rat osteogenic sarcoma cells that expressed many phenotypic features of osteoblasts. Both PTH and prostaglandins acted on their individual receptors to promote AC activity and increase cAMP formation dose-dependently in these cells (38–41). Each agonist at low concentrations rapidly activated total PKA activity (**Figure 2A**).

A notable feature seen with both PTH and PGE_2_ was that PKA activation was a much more sensitive response than that of total cell cAMP concentration (43). Activation of PKA was rapid, reaching maximum within 30 to 60 secs, with readily demonstrable activation taking place even before increases in total cell cAMP could be detected (42, 43). This is shown for PTH in osteogenic sarcoma cells and mouse calvarial osteoblasts (**Figure 2A**), with very similar data obtained with prostaglandin E_2_ (43). The data implied that only a fraction of the total amount of cAMP that can be generated by hormone stimulation is necessary to activate the protein kinase. The rapid PKA activation took place at extremely low total cell cAMP and reached its peak at agonist concentrations less than those required for maximum cAMP (**Figure 2B**). Specificity of the PKA effect was shown by its complete inhibition when the protein kinase inhibitor peptide (PKI) (44) was included in the cell isolation and incubation buffer (**Figure 2B**).

**Figure 3:**
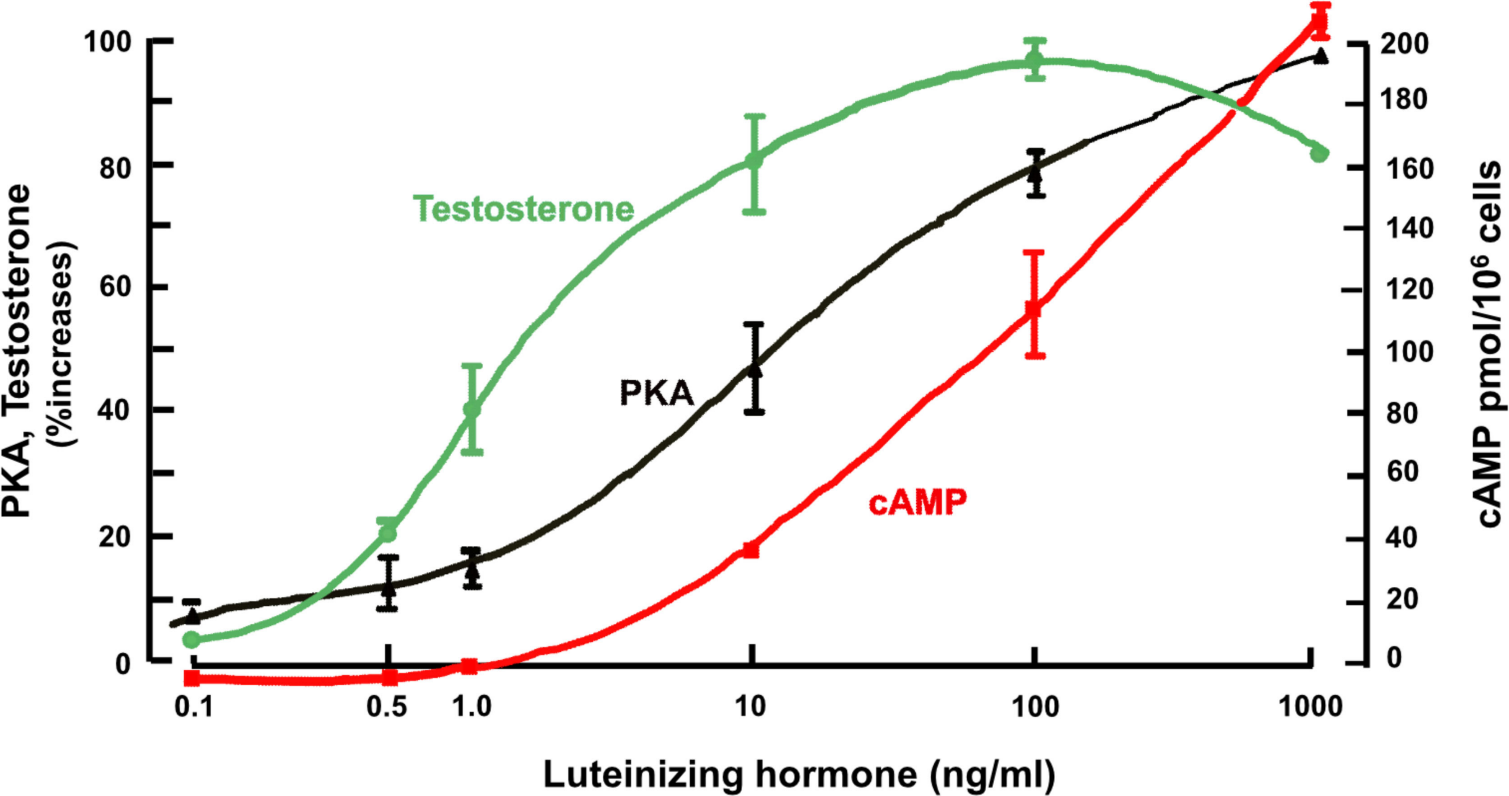
Leydig cells; effect of treatment with luteinizing hormone on PKA activity after 20 mins, cAMP and testosterone after 2 hours (from (49) with permission).

This data was similar to that obtained consistently with other hormones, including the steroidogenic hormones, human chorionic gonadotrophin (hCG) and luteinizing hormone (LH), in ovary and testis (45–47) and adrenocorticotrophin (ACTH) in adrenal (48). In the case of testis Leydig cells, for example (49), the cell-specific response of testosterone to LH increases at hormone concentrations that cause no measurable increase in cAMP, whereas the activation of PKA by LH is very closely linked to the changes in testosterone (**Figure 3**). In experiments in Leydig and adrenal cells (46) and in freshly isolated bone cells (50), cAMP occupancy of binding sites was measured in relation to total cellular cAMP generated in response to LH, adrenocorticotrophic hormone (ACTH) and PTH respectively. As cellular cAMP increased with hormone treatment, so too cAMP binding site occupancy increased. When the latter reached maximum, reflecting full activation through the PKA pathway, total cell cAMP continued to increase. These results suggest that physiological regulation of bone cell metabolism may require only modest changes in cAMP concentration, and that more cAMP is generated than is required for known specific molecular actions. For that reason, measurements of total cell cAMP might not be a reliable indicator of cell activity taking place in response to GPCR stimulation.

The conclusion from the work of these several authors was that the dissociation between cAMP and agonist response is striking and consistent, and likely to be due to extremely small changes in cAMP that transmit signals arising from activation of the receptor complex in target cells (46). With the hormone-responsive steroidogenic tissues a signaling response (steroid production) takes place relatively soon after receptor interaction and activation of PKA. On the other hand, signaling read – outs in the case of PTH1R activation of the osteoblast lineage are seen much later, and intermediate steps of regulated gene expression are poorly defined. This was the case when these studies on activation of PKA were carried out (43, 51, 52), and it remains so today.

This is relevant to the actions through the cAMP pathway that are summarized in **Figure 1**. The direct action through PTH1R to promote osteoblast differentiation and increase bone formation requires activation of many genes. The indirect effect to promote osteoclast formation requires specific actions on RANKL and OPG expression in cells of the osteoblast lineage that are located close to hemopoietic precursors. Whether the anabolic and resorptive pathways can be dissociated by altering early events in cAMP/PKA activation through PTH1R is yet to be established.

## Spare Receptors

The findings that no detectable changes in cAMP are taking place with low concentrations of agonist that nevertheless increase PKA and steroidogenesis, are consistent with the concept of “spare receptors”. This was introduced as early as 1956 in studies of muscle contractility responses and mathematical analysis of existing receptor data (53). It showed that only a small fraction of receptors needed to be occupied by agonist to elicit a maximum response. It was postulated that a given ligand can exert maximum biological effect while occupying only a small number of available receptors.

Although the concept of maximum effect being achieved without occupying all relevant receptors was at odds with earlier views of receptor occupancy proposed and held since the 1930’s, that the more receptors are activated the higher the response (54), it came to be widely accepted and applied in theory and practice to actions of drugs and of agonists for GPCRs (55). These included LH (46, 49, 56), glucagon (57), adenosine (58) and others (59). Such an effect has its counterpart for later events in spare capacity; for example hormones can generate cAMP levels greater than required for protein kinase activation, as reviewed above in the cases of PTH1R activation of osteoblastic cells (**Figure 2**) and LH receptor activation of testicular Leydig cells (49).

## Spatial Organization of Molecular Events

These aspects of early events in cAMP/PKA activation raised the possibility that the outcomes were dependent upon spatial organization and compartmentalization of components of the cAMP/PKA signaling system within cells that result in amplification of the signal. The concept developed that the isoenzymes I and II of PKA might be differentially regulated. It was thought that type II isoenzyme of PKA was involved in differentiation and growth inhibition, and type I with cell growth (60–63). An early suggestion was that membrane-associated and cytosolic protein kinases of cerebral cortex constituted subclasses of PKA isoenzyme II (64). This would provide the first level at which the general response of cAMP elevation might be channeled into specific pathways, and would provide a possible explanation for the fact that different hormones acting *via* cAMP might trigger different chains of metabolic events within the same cell.

Development of chromatographic methods of separating and analysing the individual isoenzymes led to identification that PKA isoenzymes could be individually and independently activated by isoproterenol and PGE in guinea pig and rat heart (65, 66). Modification of this approach to assess acute hormonal activation of each isoenzyme in cells in culture revealed that selective isoenzyme activation of individual isoenzymes I and II can occur in osteoblasts and osteosarcoma cells in response to PTH (43, 67), as well as in human breast cancer cells T47D and MCF7 in response to calcitonin and PGE2 (68). The cells were intact when treated but needed to be disrupted for assays, with appropriate controls. Nevertheless the mechanism of selective activation was proposed to require compartmentalization of components of the cAMP response system, even though at that stage it was not possible to assess compartmentalization directly (52).

These findings, together with increasing awareness of how the components of the cAMP/PKA signaling system interact, all suggested that what was likely to be important in post-receptor activation through the GPCR was where the cAMP was generated in relation to subsequent steps, and that total cell levels of cAMP might not reflect pharmacologic or physiologic actions.

The information regarding early actions of PTH discussed above had provided useful working conclusions concerning cell-based events in activation of the PTH receptor. Other discoveries that pointed to the possibility of compartmentalization of molecular events, and all of which applied directly to PTH action, were those of involvement of a GTP transduction process in adenylyl cyclase activation (1, 69), of a family of G proteins coupled to stimulation or inhibition of cyclase, and the discovery of β - arrestin as a regulator of GPCR internalization (70). It was quite evident however that resolution of these and other questions concerning compartmentalization of the cAMP/PKA signaling system would require development of new techniques that localized components of the system in the context of cell structure, and prevented their loss or dispersal during fixation and/or tissue preparation. Up to that time it had not been possible because of the soluble nature of some of the components (*v infra*).

## New Insights From The Identification of PTH1R AND PTHrP

**Figure 4:**
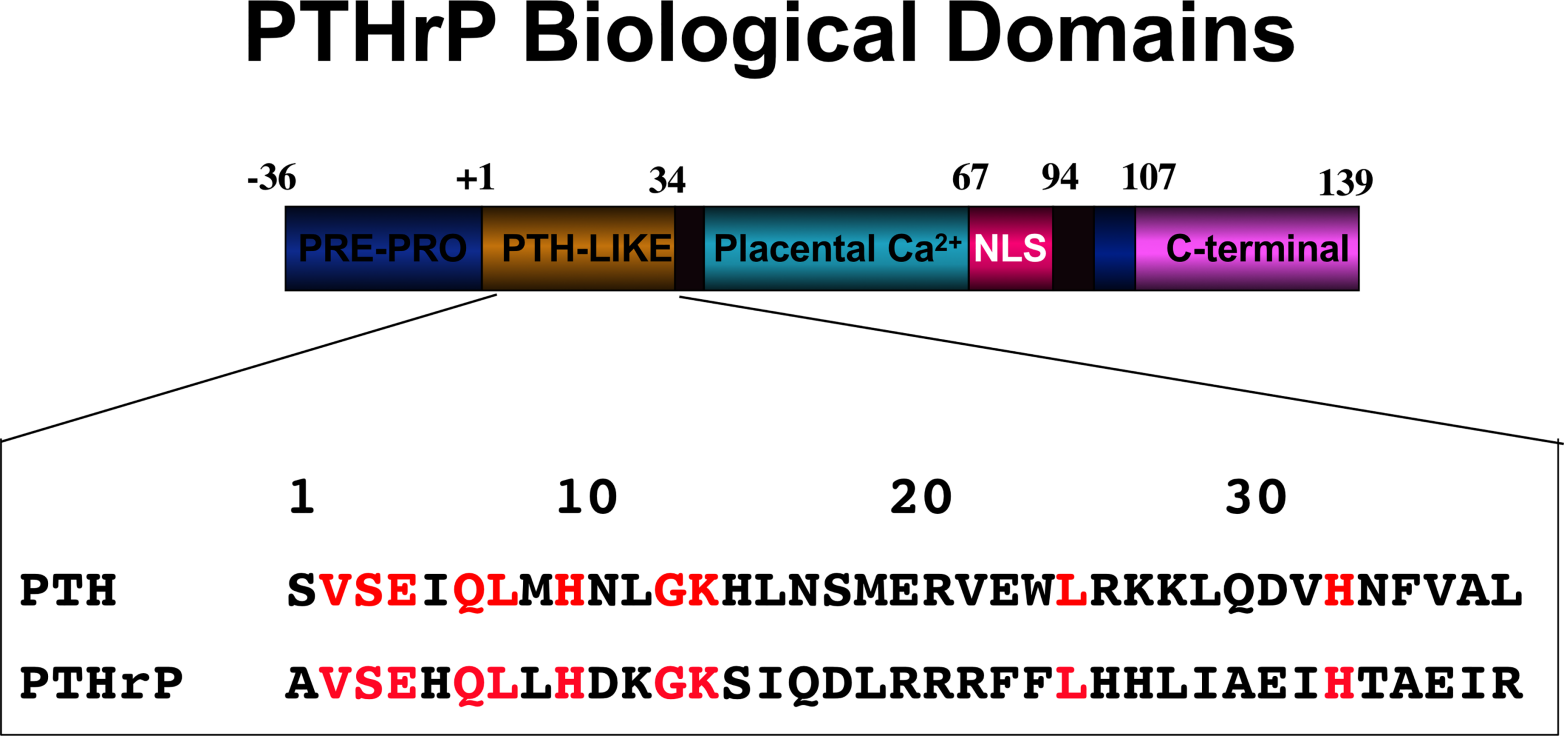
PTHrP biological domains. The amino-terminal domain of PTHrP acts like PTH upon the PTH1R. Identical residues within PTHrP and PTH are indicated in red. The other domains of action within PTHrP are indicated.

Within a few years at the end of the 1980s events developed that resulted in changes in thinking about the actions of PTH. First, the factor predominantly responsible for the humoral hypercalcemia of malignancy was identified. After it was suspected to be immunochemically similar to PTH, PTH-related protein (PTHrP) was purified, sequenced and cloned (71–73). Eight of the first 13 residues were identical to those in PTH, any remaining identities no more than expected by chance, and the structural requirements for full activation of adenylyl cyclase by PTHrP were contained within the first 34 amino acids (74), as was known to be the case with PTH (**Figure 4**). Apart from its cancer role, PTHrP could not be detected in the circulation postnatally, but was recognized physiologically as a paracrine factor in several organs, including bone (75, 76).

A second major discovery at about the same time was the cloning of the PTH receptor (PTH1R) (77), which was shown to be acted upon in a shared and equivalent manner by the amino-terminal domains of PTH and PTHrP (74, 77) in activating adenylyl cyclase activity in target cells. Accordingly, PTHrP recombinant proteins of 84, 108 and 141 (full length) residues are equipotent on a molar basis with shorter amino-terminal peptides of either PTHrP or PTH in activating adenylyl cyclase through PTH1R (78–81). Thus an unusual situation prevailed, of a circulating hormone and a paracrine factor sharing apparently equal actions upon a single GPCR.

## Endosomal Transfer of GPCR-Ligand Complexes

Identification and cloning of receptors and interacting proteins facilitated the development of reagents that could be applied using *in situ* methods to track protein spatial arrangements in cells. Before any suggestion of internalization of GPCRs, it had been shown to take place with the epidermal growth factor (EGF), a tyrosine kinase receptor. Shortly after EGF ligand addition, the majority of EGFRs and their downstream signaling factors were found located on endosomes, not on the plasma membrane, and it was concluded that EGF signaling continues from the endosomal site (82, 83). The findings with the EGF receptor provided a stimulus to look for evidence of GPCR signaling from within the cell also. When internalization of GPCRs began to be observed, early thoughts were that the receptor might engage in ß - arrestin-mediated activation of the mitogen-activated protein kinase (MAPK) pathway (84), or be degraded or recycled (85).

The new methods allowed the direct investigation of molecular events, e.g. genetically encoded fluorescent reporters that allowed direct visualization of key steps in GPCR action and cyclic nucleotide signaling, as well as fluorescent – labelled anti – receptor and anti G protein antibodies, and the facility of live cell microscopy. Such newly available methods set the scene to search for GPCR internalization and the fate of any internalized receptors.

The first published evidence showing definitively that an adenylyl cyclase – linked GPCR could continue signaling from the endosome membrane after internalization of the ligand – receptor complex came from the study of thyroid stimulating hormone (TSH) action in the thyroid follicular cell (88). Transgenic mice were used for this purpose that express a fluorescent sensor for cAMP that is expressed in virtually all cells, and in which global expression of the sensor had no effect on the phenotype of the mice. The TSH receptor internalization was found to be required to ensure appropriate expression of responsive genes.

**Figure 5:**
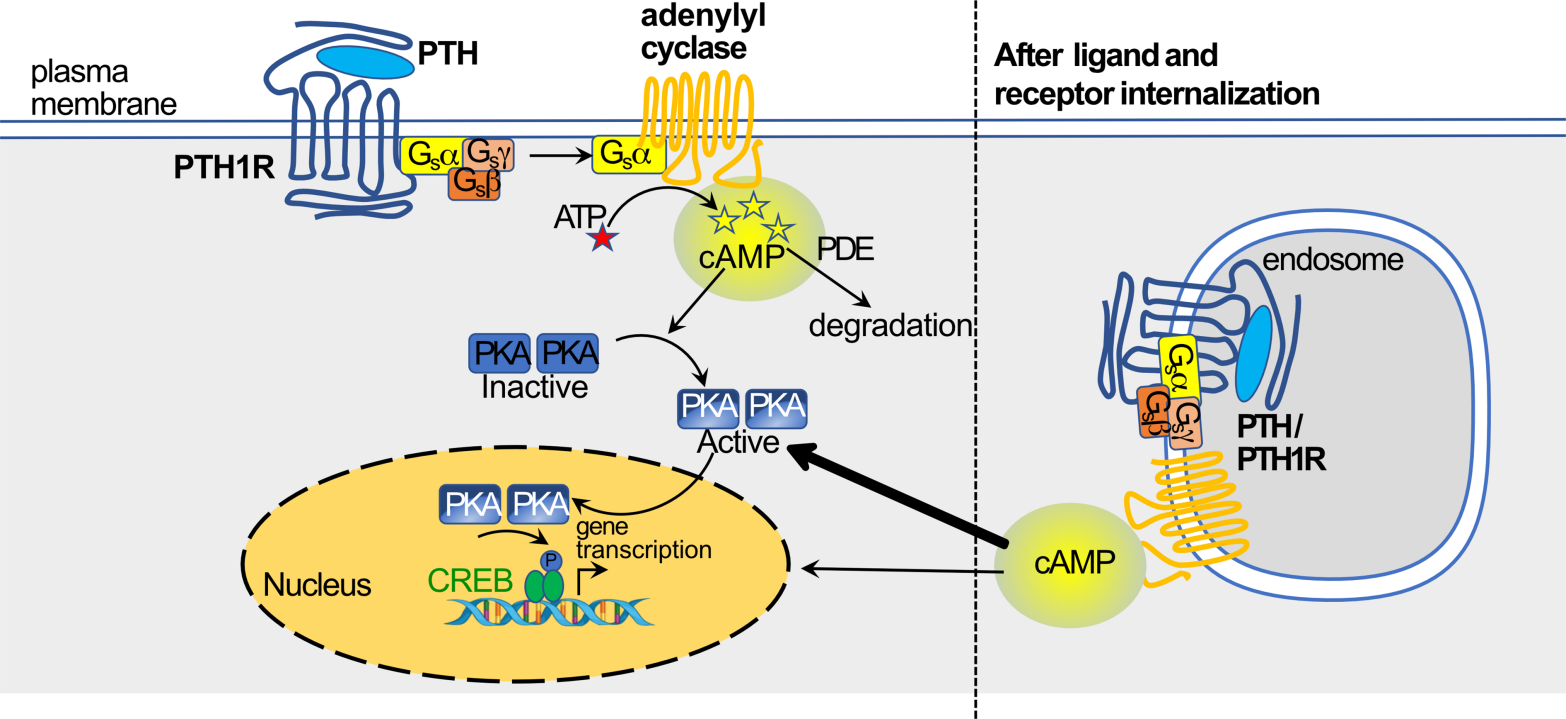
PTH activation of cAMP/PKA pathway through PTH1R, and translocation of ligand-receptor to endosome(based on work of (86, 87), figure modified from (37) with permission) (see text for details).

Demonstration of internalization of the PTH1R followed soon after. Prompted by the findings that a PTH structural analog (M-PTH) could prolong cAMP production in target cells *in vitro* and pharmacological effects *in vivo* (89), studies of protein - protein interactions and trafficking induced in cells overexpressing PTH1R showed that PTH (1–34) and PTH1R in complex were internalized to the endosome where cAMP production from adenylyl cyclase was maintained (86) (**Figure 5**). This resembled the findings of the same investigators with the vasopressin type 2 receptor, V2R (87). They showed that this persistent activation of the PTH/PTH1R complex is terminated by the endosomal retromer complex, a pentameric complex whose main function is sorting receptors away from the degradative pathway of maturing endosomes to the Golgi network (90). Such a central role of the retromer in terminating the persistent adenylyl cyclase activation by PTH/PTH1R at the endosome was confirmed by Chan et al. (91), who also provided a molecular mechanism by showing that sorting nexin 27 (SNX27) binds to PTH1R and facilitates its interaction with retromer complex. Genetic depletion of SNX27 or retromer augmented PTH1R signaling in endosomes.

These discoveries of persistent endosomal signaling *via* cAMP of hormonal agonists bound to cognate receptors brought about a radical change in thinking of the molecular mechanisms operating in the cAMP signaling pathway, with increasing evidence for spatial organization of protein – protein interactions within cells contributing to immediate and later events in target cells. The next few years saw a progressive increase in the reports of signaling through GPCRs that can continue after ligand-receptor internalization through endocytosis. These included β - adrenergic agonists (92), pituitary adenylyl cyclase activating polypeptide (93), sphingosine-1-phosphate (94), calcitonin gene-related peptide (CGRP) (95), and neurokinin 1 (96).

## Persistent Activation of Adenylyl Cyclase in Endosome by Pth And PTHrP Peptides

PTH (1–34) was already established as an approved treatment for osteoporosis in several countries (30), and PTHrP (1–36) began to be investigated clinically also because of the shared actions on PTH1R (97). Although the amino-terminal PTHrP and PTH peptides were repeatedly found to be equally potent in activating adenylyl cyclase through PTH1R in target cells (78–80), evidence was published that they might have distinct early biological activities upon receptor binding. This arose out of studies of interactions of truncated forms of PTH and PTHrP with PTH1R that led to the conclusion that PTHrP (1–36) binds to receptor, induces a brief increase in total cell cAMP and rapidly dissociates without receptor internalization (98). In addition to the binding experiments carried out in membrane preparations, PTH (1–34) in the intact cell had an effect on total cell cAMP formation that was prolonged for up to an hour by inducing PTH1R endocytosis, as a result of which PTH (1–34) – PTH1R complex continues to promote cAMP production at the endosomal level beyond the cell membrane activity (86, 87). The lack of prolonged effect on cAMP production by PTHrP (1–36) or abaloparatide was considered to be due to reduced internalization of receptor, and faster recycling possibly caused by endosomal pH sensitivity (99, 100). Abaloparatide, which had been developed as a new analog (101) for therapeutic use in osteoporosis, is identical to PTHrP in its first 21 residues, but has 8 residues different from PTHrP between 22 and 34, and is equipotent with PTH peptides in the standard bioassay of total cell cAMP generation in target cells (79, 100, 102).

An assumption grew in currency that abaloparatide promotes bone resorption less than PTH (1–34) (teriparatide), based on claims in clinical studies of greater gain in bone mineral density and greater increase in circulating bone resorption markers (31). Those claims have been called into question, based on interpretation of bone mineral density and bone marker data (32, 38, 103). It has been postulated repeatedly that these claimed differences in clinical responses can be explained by different modes of interaction with PTH1R of PTH (1–34) and abaloparatide, but no evidence for causal relationship has been obtained.

These views of differences in modes of interaction with receptor might need reconsideration in light of a further study of signaling events with peptide engagement of PTH1R. In comparing actions of PTH (1–34) and abaloparatide in an osteocyte cell line, a number of assays were used that showed that PTH (1–34) and abaloparatide were indistinguishable in their early receptor-related effects (102). They induced intracellular calcium equally and were equipotent in a standard cAMP response assay with full phosphodiesterase inhibition, as has been shown many times (100, 104). When PTH (1–34) and abaloparatide were each labelled with a fluorophore and tracked after 10 nM treatment of PTH1R +ve cells, both were effectively internalized, and they had indistinguishable dose-responsive effects on internalization of a fluorescent-labeled PTH1R. Since β - arrestin recruitment was known to be required for endosomal GPCR signaling this was examined, and abaloparatide and PTH (1–34) induced β - arrestin clustering to the same extent and clearly more than a negative control. Although PKA pathway activation was not measured directly, the PKA pathway was assessed by using a degenerate phospho – specific antibody to detect outcome of PKA activity. This revealed no difference in phosphorylation of substrates between the two ligands, PTH (1–34) and abaloparatide. Further, PTH (1–34), abaloparatide and M-PTH showed comparable effects on salt inducible kinase (SIK2) phosphorylation, substrate dephosphorylation and downstream gene expression (102). All of these findings suggest equivalent signaling effects of PTH (1–34) and abaloparatide.

The authors of this study (102) indicated that their findings of no subtle differences between PTH (1–34) and abaloparatide in early actions that have been previously reported (98–100) might relate to limitations in design and sensitivity of assays. They also consider the possibility that the differences noted by others in clinical studies might be related to pharmacokinetic or other factors (102). The questions nevertheless remain (i) whether there are consistent differences in action between PTH on the one hand and either abaloparatide or the N-terminal domain of PTHrP on the other, and (ii) whether significant differences in pharmacological effects exist between PTH and abaloparatide (103). Thus although the endosomal transfer of PTH/PTH1R complex resulting in persistent endosomal cAMP generation is now well established (**Figure 5**), the results of Sato et al. (102) call for reappraisal of the comparisons that have been made between the molecular events in response to PTH (1–34) and those in response to abaloparatide. A similarly reappraised comparison of PTH (1–34) and PTHrP (1–36) action would also be helpful.

Despite these similar actions of PTH (1–34) and abaloparatide, it has nevertheless been shown in another study that it is possible to modify PTH structurally to separate plasma membrane from endosomal activation. An analog of PTH prepared by epimerization of residue 7 (PTH^7d^) behaved as a biased agonist, eliciting cAMP production at the plasma membrane but not at the endosome (103). In *in vivo* studies in which bone formation was not measured, PTH^7d^ treatment had no effect on 1-hydroxylation of vitamin D, whereas a long acting PTH peptide that shows endosomal transfer and persistent cAMP effect much greater than PTH (1–34) (89), substantially increased vitamin D 1-hydroxylation and the amount of bone assessed by microcomputed tomography (103). This further supports a biological role for endosomal PTH action, but unfortunately whether this might contribute to either an anabolic or a resorptive effect of PTH was not addressed. Such differences from PTH (1–34) in pharmacological response would need to be shown with PTHrP (1–36) and abaloparatide if any differences in signaling are sufficient to influence later tissue outcomes. The question of whether small changes in duration of cAMP elevation through endosome-translocated PTH1R complex can influence later, major consequences of PTH1R activation, has not been investigated.

As described above, there is compelling evidence that prolongation of activation of PTH1R, e.g. by repeated or high dose injection or infusion, results in conversion of an anabolic to a resorptive response (35, 36, 104). On present evidence the same cannot be said of the changed dynamics in total cell cAMP levels that can accompany endosomal translocation of the PTH/PTH1R complex (86, 98), or that have been ascribed to treatment of cells with abaloparatide (100).

## Pharmacologic Significance of Persistent Endosomal Generation of cAMP

Specific, later phenotypic responses in cells have been related directly to continued endosomal signaling in the cases of several GPCRs. These include β - adrenergic agonists (92), TSH (105), pituitary adenylyl cyclase activating peptide (93), calcitonin gene-related peptide (95) and neurokinin -1 (96). This has yet to be done with the PTH1R. Most attention has been focussed upon PTH and PTHrP peptides used therapeutically, and findings do not necessarily relate to physiology. This is an important distinction, since PTH, a circulating hormone, shares actions upon a common receptor, PTH1R, with the paracrine/autocrine agent, PTHrP.

In *in vitro* studies the only identifiable form of PTHrP released by osteocytes and capable of activating PTH1R was found to be full length PTHrP (106). This mechanism for secretion of PTHrP in those cells of mesenchymal origin is consistent with the evidence that many cells, including those of mesenchymal origin (osteoblasts included) secrete proteins by a constitutive mechanism, rather than the regulated pathway that packages and processes proteins to daughter peptides before their secretion (107).

Since PTHrP acts in a paracrine manner, effects on PTH1R activity of brief exposure to PTHrP of varying lengths and to PTH were studied. Brief exposure to full length PTHrP in several osteoblastic cell culture systems, followed by washout of the cells, resulted in activation of adenylyl cyclase that persisted for some hours after ligand washout. This effect was not seen with shorter PTHrP peptides or with PTH peptides. This persistent activation by PTHrP (1–141) was found also with the long acting analog M-PTH, and in both cases the persistent response following brief exposure and washing of the cells was associated with prolonged activation of CRE-luciferase and regulation of osteoblastic genes that was seen up to 24 hours later (108). Although direct demonstration of PTHrP/PTH1R translocation was not sought in this work, the effects of PTHrP were blocked by two pharmacological inhibitors of endosomal transfer, Dyngo and Pitstop (108), suggesting that endosomal translocation had taken place and was required for these effects. Although this falls short of identifying an end response of an anabolic or resorptive action through the PTH1R (see **Figure 1**) in relation to endosomal activity, it does illustrate that that prolongation of PTH1R-related action through cAMP/PKA can influence later gene expression in these target cells. This might be relevant to the effects on bone seen with *in vivo* injection of M-PTH (89). Such stimulation after brief exposure might be a property particularly suitable for an autocrine/paracrine effector such as PTHrP acting through PTH1R in bone. After local generation it would be expected to be exposed briefly to target cells.

## Conclusion and Summary

Activation of the PTH1R in osteoblast lineage cells through the cAMP/PKA signaling pathway results in either of two effects *in vivo*, promotion of bone formation or of bone resorption. The formation effect requires action upon several stages of the osteoblast lineage to promote gene expression and differentiation within the lineage. The resorption effect requires regulation through PTH1R of RANKL and OPG in osteoblast lineage cells that have close access to hemopoietic precursors of osteoclasts (as depicted in **Figure 1**).

The finding that PTH1R can be activated in a manner that achieves maximum effect without occupying all receptors is similar to that with other GPCR agonists. It has yet to be shown with any PTHrP preparations, but the action shared with PTH on PTH1R makes it likely. Such a mechanism could help compensate for low molecular numbers, and could provide a target for development of superagonist compounds (59). For example low PTH1R receptor numbers might contribute to the findings in cell tracking experiments that functional PTH1R is expressed earlier in cells of the osteoblast lineage than previously suspected (109). Despite low receptor numbers in the early osteoblast precursor cells, this could present as a pharmacological target to enhance osteoblast differentiation.

There is much interest in understanding the processes involved in the distribution of GPCR activation between the plasma membrane and the endosome, and the significance of this for later events. For example, chemically distinct ligands of the β - adrenoreceptor can either be selective for plasma membrane activation only or for endosomal activation also. The endosomal activation was found to be needed for the full repertoire of downstream cAMP/PKA effects (92). Later work (110, 111) accentuated the functional contribution of endosomal cAMP production to the phosphorylation patterns in target cells. If such information could be gained for PTH1R activation insights from that could indicate new directions in therapeutic approaches through the PTH1R. Such information could be very informative in designing new ways of using the PTH1R in therapeutic approaches.

## Author Contributions

The author confirms being the sole contributor of this work and has approved it for publication.

## Conflict of Interest

The author declares that the research was conducted in the absence of any commercial or financial relationships that could be construed as a potential conflict of interest.

## Publisher’s Note

All claims expressed in this article are solely those of the authors and do not necessarily represent those of their affiliated organizations, or those of the publisher, the editors and the reviewers. Any product that may be evaluated in this article, or claim that may be made by its manufacturer, is not guaranteed or endorsed by the publisher.
